# Knowledge on preconceptional folic acid supplementation and intention to seek for preconception care among men and women in an urban city: a population-based cross-sectional study

**DOI:** 10.1186/s12884-015-0774-y

**Published:** 2015-12-18

**Authors:** Sevilay Temel, Özcan Erdem, Toon A. J. J. Voorham, Gouke J. Bonsel, Eric A. P. Steegers, Semiha Denktaş

**Affiliations:** Department of Obstetrics and Gynaecology, Division of Obstetrics and Prenatal Medicine, Erasmus MC, Westzeedijk 118, Room Wk-221, 3016 AH Rotterdam, The Netherlands; Municipal Health Service Rotterdam-Rijnmond, Rotterdam, The Netherlands; Department of Obstetrics and Gynaecology, Division of Obstetrics and Prenatal Medicine, Department of Public Health, Erasmus MC, Rotterdam, The Netherlands

**Keywords:** Preconception Care, Folic Acid, Intention, Health professional, Knowledge

## Abstract

**Background:**

To study the knowledge of a large city population on preconception folic acid supplementation and intention to seek for preconception care within an urban perinatal health program.

**Methods:**

Cross-sectional surveys run in Rotterdam, the Netherlands, in 2007 and annually from 2009 to 2014. A random sample of residents aged between 16 and 85 years was taken each year from the municipal population register. Bivariate analysis, interaction analysis, trend analysis and logistic regression were performed.

**Results:**

Knowledge on preconceptional folic acid supplementation significantly improved (+20 %) between 2007 and 2009, and the intention to consult a GP or midwife in the preconception period significantly increased (+53 %) from 2007 to 2012. Logistic regression analyses showed that low socio-economic status was significantly associated with low preconceptional folic acid knowledge, but with higher intention to seek out preconception care. An interaction effect was found between educational level and ethnicity, showing that the higher the educational level the lower the gap of level of knowledge between the different ethnic groups.

**Conclusion:**

Despite campaigns about folic acid supplementation knowledge on this supplement remains low. The intention amongst men and women to seek out preconception care is still insufficient. Structural interventions to increase and maintain awareness on folic acid supplementation, especially among high-risk groups, are needed.

## Background

Preconception care (PCC) is an essential component of maternal and child healthcare. PCC is defined as the set of preventive interventions targeted at women of reproductive age and their partners to improve pregnancy outcomes. A wide range of preconception risk factors are found to be associated with adverse foetal outcomes, and many of these risk factors are amenable to prevention [1, 2]. For selected outcomes (e.g. neural tube defects (NTDs)), prevention through PCC have shown to be effective [3].

A population study showed that 98 % of couples hoping to have a child exhibit at least one risk factor amenable for intervention, thus justifying individual counselling [4]. Despite the seemingly straightforward positive benefits of PCC and the high percentage (80 %) of planned pregnancies (among the native Dutch population) fit for the application of PCC [5], the observed number of individual PCC consultations is negligible [6]. Lack of knowledge about common preconception risk factors seems to be one of the critical factors hindering the widespread application of PCC: in a population study in Rotterdam half of the non-pregnant study population (*n* = 631) were unaware of the adverse effect of smoking and being overweight on fertility [7]. Although, this outcome is in contrast with other results [8], specific preconception health knowledge, e.g. folic acid (FA) use, was also scarce [7].

Since appropriate FA use before and during early pregnancy has been shown to protect against NTDs and other congenital anomalies [9, 10], public awareness campaigns have been launched to raise FA supplementation [11]. In the Netherlands, the last government-sponsored mass-media campaign was in 1995. In absence of continued reinforcement of this message, the use of FA supplementation has lowered. The initiative of local pharmacies in 2004 to inform women using oral contraceptives of the benefits of preconceptional FA supplementation seemed to have had little effect on FA use, with supplementation levels remaining at 36 % [12].

**Fig. 1:**
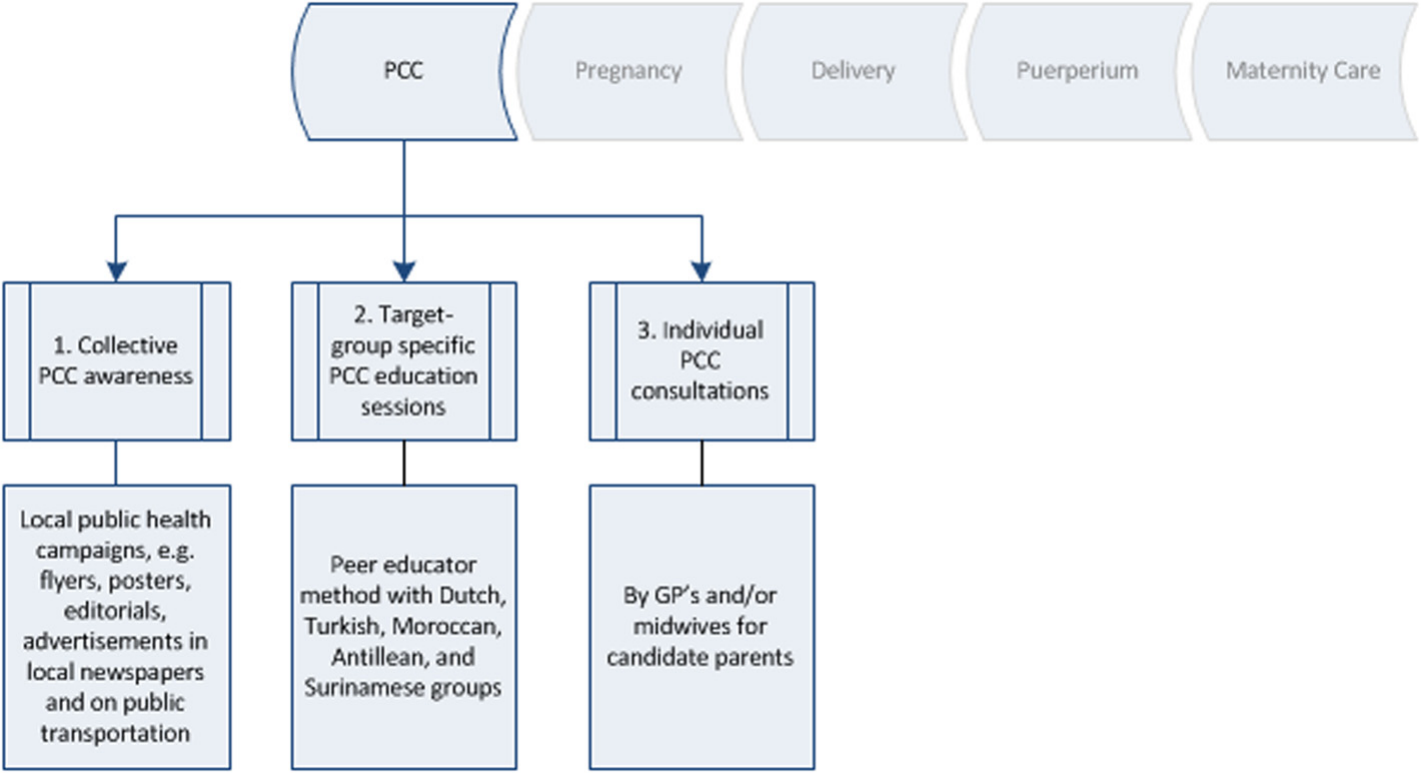
Obstetrical chain of care within the Ready for a Baby program. The PCC sub-study was the first chain in this comprehensive perinatal health program. The objective of the PCC sub-study was to develop and organize standardized general PCC. For this purpose three approaches were combined in two pilot districts of Rotterdam: (*1*) collective PCC awareness through local public health campaigns, (*2*) target group-specific PCC education sessions through the ‘peer educator’ method, and (*3*) individual PCC consultations by GP’s and midwives

In 2009, the Rotterdam municipality and the Erasmus University Medical Centre, supported by health scientists, launched the Ready for a Baby program [13] aimed at improving perinatal health. The PCC sub-study was the first chain in this comprehensive urban program, including PCC pilot projects in selected North (2008–2010) and South (2010–2012) districts of the city of Rotterdam. Beyond interventions to raise awareness for preconceptional FA use and PCC utilization, the program aimed at reaching vulnerable population groups, such as migrants and those with low socio-economic status (Fig. 1).

The aim of the present study was to investigate knowledge of preconception FA supplementation and intention to seek for PCC in the city of Rotterdam.

## Methods

### Study design

Within the PCC sub-study three pillars (Fig. 1) were combined to have maximum reach and impact: 1) the aim of the public campaign was to raise awareness and attention about the provision and the content of PCC, and to provide information about activities in the PCC pilot projects. 2) The aim of the target groups-specific PCC sessions was to transfer knowledge and awareness about PCC and to motivate the target group to make use of individual PCC. Three types of education were developed and executed by six women and one men from different ethnic origin given in their native language through their existing network in the neighbourhoods and places where people met each other, e.g. mosques and schools. The course centered around information provision about the importance of a good preparation of a pregnancy, the timely intake of FA supplementation, prenatal screening, and the prevalence of genetic disorders among some migrant groups, such as sickle cell disease and Thalassemia with several materials to make the sessions interactive. On the side note other health promotion approaches were also involved like enhancing autonomy and empowerment, but the effects of these other approaches on the results were not measured in this study. In the final session, the group of women accompanied by the peer educator visited a midwifery practice in the neighbourhood.

The men’s session was compromised to a single session. 3) General individual PCC was provided by GP’s and midwives using www.ZwangerWijzer.nl; an evidence-and practice-based web application that for candidate parents allows to make an individual risk assessment [14].

**Fig. 2:**
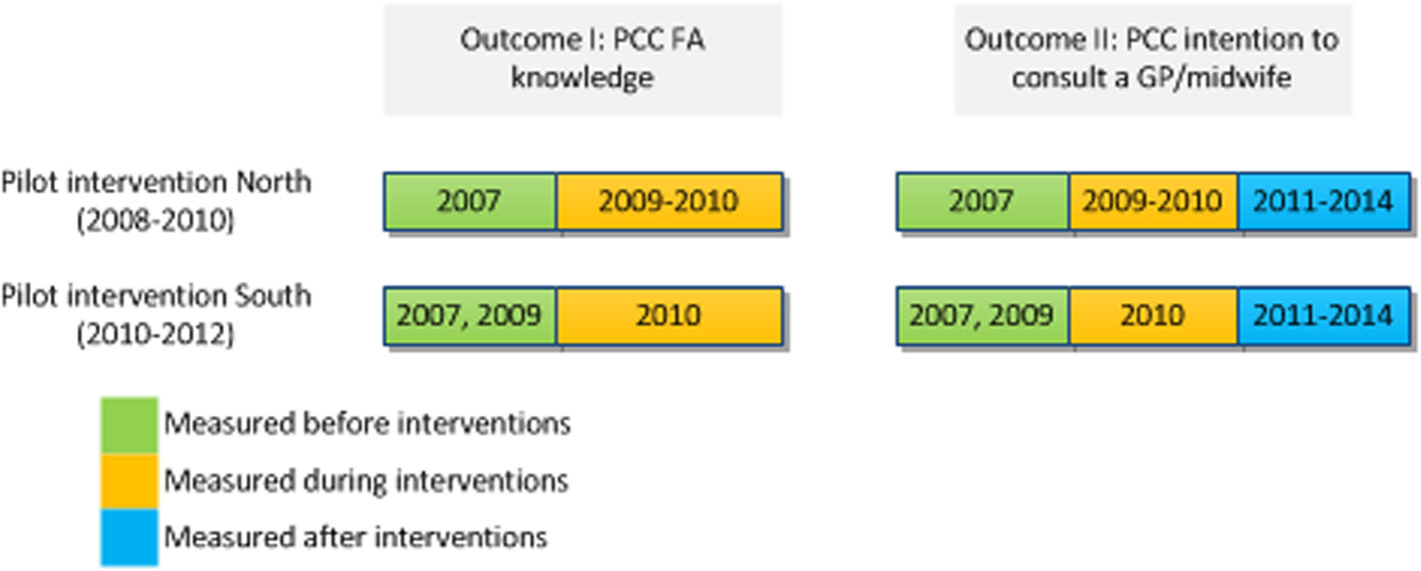
Outcomes within the Omnibus Survey per pilot intervention district and measured study years correlating with the pilot interventions

In this population-based study we used seven citywide cross-sectional surveys (2007, 2009–2014) which measured citywide and two districts knowledge on preconception FA supplementation and intention to seek out PCC at different times. Using these seven cross-sectional surveys we performed a trend-analysis to observe changes in PCC knowledge. See Fig. 2 for a scheme of the study design.

### Outcomes

The primary outcome was preconception FA knowledge measured over 3 years (2007, 2009, and 2010). This outcome reflected the affirmative response to the question ‘A woman who wishes to become pregnant should take FA supplementation before she tries to become pregnant’, with three non-ordered response levels (no, that is not necessary; yes, she certainly should; and don’t know/ not relevant). After 2010 this outcome was not included in the survey due to different policy prioritization.

The secondary outcome was the response to the question regarding the intention to seek out PCC, measured over seven consecutive years (2007, 2009–2014). This outcome reflected the response to the question ‘A woman who wishes to become pregnant should consult a GP or midwife before she tries to become pregnant’, with four non-ordered response levels (no, that is not necessary; only in case of problems; yes, she certainly should; and don’t know/ not relevant). In 2008 both outcomes were not included in the survey.

In terms of medical adequacy (first outcome) and given that every couple could benefit from PCC (second outcome), ‘*yes, she certainly should*’ was regarded as the only correct response. Response levels on both outcomes were recoded into a two-level outcome (‘other’ vs. ‘yes’) in order to perform logistic regression analysis.

### Population

Rotterdam is the second largest city in the Netherlands (610,412 inhabitants: 48 % non-Dutch origin) with the highest number of deprived neighbourhoods (seven) and large socio-economic inequalities [15]. The four major immigrant groups are: Surinamese 8.5 %; Turkish 7.8 %; Moroccan 6.7 %; and Antillean 3.8 % [16]. Although the ethnic minorities and the age category <45 years are underrepresented in the Omnibus Survey, the response rate is still representative for the whole city, and large enough to make statements and search for changes in trends [17]. The age category over 45 years was included in the analysis as knowledge of elderly family members about PCC can influence the behaviour of younger people within their family [18]. Although the existing of cultural and socio-economic differences relating to responsibility and family planning, men were also included. Their role in PCC is important: family planning is a shared responsibility and some risk factors, such as smoking and sexually transmitted diseases, are joint [19]. Moreover, literature on gender differences in PCC utilization is scarce.

### Data collection

Study-specific survey items were added to the *Omnibus Survey*, a paper survey run annually using a random sample of 3500 Rotterdam citizens aged between 16 and 85 years. The study-specific items were previously tested and included in a survey which was used to study the determinants of the intention of PCC use [7]. In case of insufficient response among the immigrant groups, trained male and female interviewers administered the Dutch questionnaire face-to-face in order to achieve representativeness. The percentage of these face-to-face interviews fluctuated between 2.2 and 8.6 % of the total study sample per year. The response rate (including additional face-to-face interviews) between 2007 and 2014 gradually decreased from 42 % (*n* = 1329) to 29 % (*n* = 897) (Table 1). Explanations for this decrease could be that Dutch language proficiency acts as a barrier to fill out the written survey and/or big city’s population are less receptive to forms of social participation, such as participating in a municipal survey.

**Table 1:** Study size and response rate per study year in the Omnibus Survey

Study year^e^	PCC FA knowledge^a^		PCC intention to consult a GP/midwife^b^	
	Response size^d^, *n*	Response rate, %	Response size^d^, *n*	Response rate, %
2007	1314	38	1329	42
2009	1240	35	1250	40
2010	1212	35	1225	38
2011	NA^c^	NA^c^	1194	38
2012	NA^c^	NA^c^	1105	37
2013	NA^c^	NA^c^	1080	35
2014	NA^c^	NA^c^	897	29

^a^Preconceptional folic acid knowledge based on: “A woman who wishes to become pregnant should take folic acid supplementation before she tries to become pregnant”. Rotterdam, the Netherlands, 2007, 2009 and 2010

^b^Preconceptional intention to consult a GP or midwife based on: “A woman who wishes to become pregnant should consult a GP or midwife before she tries to become pregnant”. Rotterdam, the Netherlands, 2007, 2009–2014

^c^ *NA* Not accounted

^d^Response size represents the number of returned and filled out surveys

^e^Each study year the study sample covered 3500 participants aged 16–85 drawn from the municipal register

### Independent variables

*Time in years* corresponding with before, during and after the pilot campaigns. *Ethnicity* was based on the country of birth of the individual and his/her parents as registered in the civil administration. The different ethnicities were subsequently recoded into three categories: (1) non-Western immigrants (e.g. Moroccan, Turkish, Antillean, Surinamese, Cape Verdean, Aruban, Asian, and African); (2) Western immigrants (e.g. European and American); and (3) Dutch.

*Educational level*, *household income*, *employment status*, and *neighbourhood* were used as indicators of socio-economic status. *Educational level* was determined on the basis of the highest completed education (no education/primary education, lower secondary education, higher secondary education, and higher vocational college/university) and classified into two categories: 1) low and; 2) moderate and high. *Household income* reflected monthly income and was divided into 6 categories (<950 euro, 950–1300 euro, 1300–1900 euro, 1900–3150 euro, 3150–3500 euro, and 3500 euro and more) being consecutively adjusted for the number of persons in the household and classified into 1) minimum; 2) minimum-moderate; 3) moderate-2× moderate; and 4) >2× moderate. To determine *employment status* respondents were asked whether they had paid work and their responses were recoded into: 1) unemployed and; 2) employed. Respondents were categorized as residing in or outside a deprived *neighbourhood* on the basis of the postal code of their place of living derived from the Government Decision of May 2007 [20].

The variable *children living in the household* was measured by asking how the household was composed (alone, two adults with no children in household, (married) couple with children in household, and one parent with children in household) and was classified into 1) no and 2) yes.

*Religion* was measured by asking the respondents whether they considered themselves as belonging to a religion; answers were recoded into: 1) no and; 2) yes.

### Data analysis

A Chi-Square test (*X*^2^-statistics) was used for bivariate analysis of correct preconceptional FA knowledge and correct knowledge regarding intention to seek out PCC (*p*-values <0.05 were regarded statistically significant). Using Spearman’s rank correlation, no coefficient association (r > 0.60) was found between both outcomes. Trend analysis for changes in knowledge of preconception FA supplementation and PCC consultation was performed for both pilot districts separately as well as citywide (including the two pilot districts). The logistic regression analysis was performed in a two- and three-stage approach: in model I the study years were entered, in model II socio-demographics, socio-economic status and other variables were included, and in model III the observed interaction between ethnicity and educational level was included. Results are reported as (adjusted) odds ratios (OR), with 95 % Confidence Intervals (CI).

### Details of ethical approval

The research proposal has been reviewed by the Medical Ethics Review Committee of the Erasmus Medical Center. As a result of this, the Committee informed us that the rules laid down in the Medical Research Involving Human Subjects Act (also known by its Dutch abbreviation WMO), do not apply to this study as data collection was anonymous and no invasive treatments were performed. It was therefore not necessary to obtain informed consent.

## Results

Characteristics of the study population and percentages of correct answers for both outcomes are shown in Table 2. Significantly more correct answers for the preconception FA questions were given by those in the following respondent categories: females, those aged between 25 and 44 years old, Dutch respondents, individuals with a moderate and high educational level, those with income more than two times the moderate household income, employed respondents, respondents living in households with children, and non-religious respondents. Significantly more correct answers for the intention to seek out PCC were given by the following respondent categories: males, non-Western respondents, individuals with a low educational level, those with a minimum household income, unemployed respondents, respondents living in deprived neighbourhoods, and those living in households with children.

**Table 2:** Characteristics of the study population in the Omnibus Survey

	PCC FA knowledge^a^ (*n* = 3766)	Correct answer (%)	*p*-value*	PCC intention to consult a GP/midwife^b^ (*n* = 8080)	Correct answer (%)	*p*-value*
Socio-demographics						
Gender, %			<0.001			<0.001
Men	45	26		45	21	
Women	55	43		55	18	
Age in years, %			<0.001			0.18
16–24	11	24		11	22	
25–44	33	51		33	19	
≥ 45	56	27		56	19	
Ethnicity, %			<0.001			<0.001
Non-Western immigrants	24	29		23	26	
Western immigrants	9	35		9	21	
Dutch	67	37		68	17	
Socio-economic status						
Educational level, %			<0.001			0.02
Low	42	27		40	21	
Moderate and high	55	41		60	19	
Household income, %			<0.001			<0.001
Minimum	22	25		24	25	
Minimum-moderate	22	29		22	22	
Moderate-2× moderate	28	37		31	16	
> 2× moderate	22	47		23	16	
Employment status, %			<0.001			<0.001
Unemployed	49	27		46	21	
Employed	51	42		54	18	
Neighbourhood, %			0.22			<0.001
Non-deprived	67	36		66	18	
Deprived	33	34		34	22	
Other variables						
Children living in the household, %			<0.001			0.02
No	60	29		63	20	
Yes	40	43		37	18	
Religion			0.003	NA		NA
No	52	37				
Yes	48	32				

*p-value for differences between correct answers

^a^Preconceptional folic acid knowledge based on: “A woman who wishes to become pregnant should take folic acid supplementation before she tries to become pregnant”. Rotterdam, the Netherlands, 2007, 2009 and 2010

^b^Preconceptional intention to consult a GP or midwife based on: “A woman who wishes to become pregnant should consult a GP or midwife before she tries to become pregnant”. Rotterdam, the Netherlands, 2007, 2009–2014

**Fig. 3:**
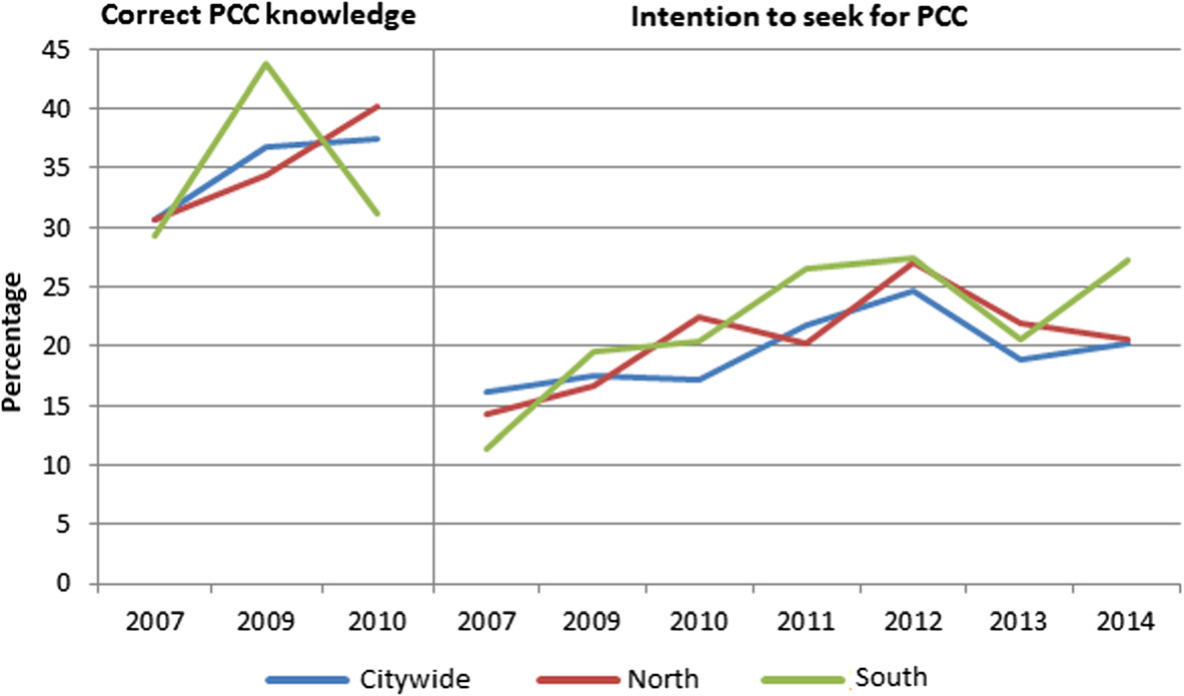
Trend analysis for correct PCC knowledge and intention to seek for PCC. Trend analysis for the city of Rotterdam and focusing on the pilot districts North and South (2007, 2009–2014)

Trend analysis (Fig. 3) showed that the overall correct knowledge of preconceptional FA supplementation significantly increased from 30.7 % in 2007 to 36.8 % in 2009 (*p* = 0.001), with no further increase in 2010. For district North (*n* = 298; response rate 36.9 %) a non-significant increase of 31 % was found between 2007 (30.6 %) and 2010 (40.2 %). For district South (*n* = 103; response rate 9.8 %) a fluctuating pattern was found, with a non-significant increase from 29.3 % in 2007 to 43.7 % in 2009, followed by a non-significant decrease to 31.2 % in 2010.

The overall intention to seek out PCC significantly increased from 16.1 % in 2007 to 24.6 % in 2012 (*p* < 0.001), with a temporary decrease to 18.8 % in 2013 and an increase to 20.3 % in 2014. For district North (*n* = 694; response rate 34.8 %) a non-significant increase was observed between 2007 (14.3 %) and 2010 (22.4 %), with a fluctuating pattern afterwards. For district South (*n* = 711; response rat 28.6 %) a significant increase was observed between 2007 (11.3 %) and 2012 (27.4 %), with a fluctuating pattern exhibiting decreases and increases after 2012.

Results from the logistic regression analysis for both outcomes with ‘yes, she certainly should’ as reference category are shown in Table 3. Knowledge of preconception FA supplementation was significantly lower in: the study year 2007, in men, in persons aged between 16 and 24 years or 45 years and older, in persons with a non-Western immigrant background, in minimum and minimum-moderate income households, and in households without children. The intention to seek out PCC was significantly lower in the study years 2007, 2009, and 2010. Significantly higher PCC intention was found in: the study year 2012, in men, in persons aged between 16 and 24 years, in persons with an immigrant background, in minimum and minimum-moderate income households, and in households without children.

**Table 3:** Results from logistic regression analysis for preconceptional folic acid knowledge and preconceptional intention to seek for care with “yes, she certainly should” as reference category

	Preconceptional folic acid knowlegde^a^			Preconceptional intention to seek for care^b^	
	I^c^	II^c^	III^c^	I^c^	II^c^
Study years					
2007	0.74 (0.63–0.87)**	0.73 (0.61–0.88)**	0.73 (0.61–0.88)**	0.53 (0.40–0.69)**	0.51 (0.39–0.68)**
2009	0.97 (0.82–1.14)	1.00 (0.83–1.19)	0.99 (0.83–1.19)	0.61 (0.47–0.81)**	0.61 (0.46–0.81)**
2010	reference	reference	reference	0.58 (0.44–0.77)**	0.61 (0.46–0.81)**
2011	NA	NA	NA	1.14 (0.86–1.51)	1.19 (0.89–1.59)
2012	NA	NA	NA	1.42 (1.07–1.90)*	1.54 (1.14–2.07)*
2013	NA	NA	NA	1.00 (0.75–1.34)	1.01 (0.75–1.37)
2014	NA	NA	NA	reference	reference
Socio-demographics					
Gender (reference: women)					
Men		0.42 (0.36–0.49)**	0.42 (0.36–0.49)**		1.41 (1.22–1.64)**
Age in years (reference: 25–44)					
16–24		0.31 (0.24–0.40)**	0.30 (0.23–0.39)**		1.38 (1.07–1.79)*
45 and older		0.40 (0.34–0.48)**	0.40 (0.33–0.47)**		1.09 (0.91–1.30)
Ethnicity (reference: Dutch)					
Non-Western immigrants		0.57 (0.46–0.71)**	0.45 (0.33–0.62)**		1.94 (1.59–2.35)**
Western immigrants		0.69 (0.53–0.91)*	0.74 (0.44–1.26)		1.59 (1.23–2.05)**
Socio-economic status					
Educational level (scale)		1.31 (1.10–1.55)*	1.20 (0.98–1.47)		1.13 (0.94–1.35)
Household income (reference: >2× moderate)					
Minimum		0.59 (0.46–0.77)**	0.60 (0.46–0.77)**		1.56 (1.22–2.01)**
Minimum-moderate		0.66 (0.53–0.83)**	0.66 (0.52–0.82)**		1.60 (1.26–2.02)**
Moderate-2× moderate		0.79 (0.65–0.96)*	0.78 (0.64–0.95)*		1.07 (0.87–1.31)
Employment status (reference: employed)					
Unemployed		0.93 (0.78–1.10)	0.92 (0.78–1.10)		1.18 (1.00–1.40)
Neighbourhood (reference: deprived)					
Non-deprived					0.97 (0.83–1.15)
Other variables					
Children living in the household (reference: yes)					
No		0.60 (0.51–0.71)**	0.60 (0.51–0.70)**		1.60 (1.36–1.88)**
Religion (reference: yes)					
No		1.03 (0.88–1.21)	1.03 (0.87–1.20)		NA
Interaction					
Ethnicity*educational level (ref: Dutch* educational level)					
Non-Western* educational level			1.46 (1.01–2.11)*		
Western* educational level			0.92 (0.50–1.70)		

^a^Preconceptional folic acid knowledge among respondents answering ‘other’ vs. ‘yes’ on: “A woman who wishes to become pregnant should take folic acid supplementation before she tries to become pregnant”. *N* = 3766; Rotterdam, the Netherlands, 2007, 2009 and 2010

^b^Preconceptional intention to consult a GP or midwife among respondents answering ‘no’ vs. ‘yes’ on: “A woman who wishes to become pregnant should consult a GP or midwife before she tries to become pregnant”. *N* = 2771; Rotterdam, the Netherlands, 2007, 2009–2014

^c^In model I the study years were entered, in model II socio-demographics, socio-economic status and other variables were included, and in model III the observed interaction between ethnicity and educational level was included

Data are in OR, odds ratio (95 % confidence interval). OR = exp (ß), where ß is the coefficient in the logistic model

* p ≤0.05; ** p ≤0.001

*NA* Not Accounted

Nagelkerke for preconceptional folic acid knowledge: step I R^2^ = 0.006, step II R^2^ = 0.181, step III R^2^ = 0.181 and for preconceptional intention to seek for care: step I R^2^ = 0.042, step II R^2^ = 0

### Analysis of interaction between determinants

Regarding the outcome preconceptional knowledge of FA supplementation, one interaction was found between ethnicity and educational level. The knowledge gap between non-Western immigrants and the Dutch was significantly larger amongst individuals with low levels of education. This gap was not apparent among individuals with high levels of education. No significant interactions were observed between gender and the variables: age, educational level, employment status, neighbourhood, having children in the household, and religion.

## Discussion

The present study demonstrates that correct knowledge concerning both FA supplementation (30.7 % in 2007 vs. 36.8 % in 2009) and the need for PCC consultation before pregnancy (16.1 % in 2007 vs. 24.6 % in 2012) significantly increased citywide during the study years. . Non-significant changes in districts North and South where the interventions took place were observed. With a public health approach via the Ready for a Baby program in the North and South districts of Rotterdam interventions were set up to raise awareness for preconceptional FA use and PCC utilization. These interventions may have influenced knowledge in the two intervention districts. Although, representativeness of the study sample is good enough for generalizability and validity, still it remains difficult to measure effects citywide from interventions executed in a part of the city. Moreover, measuring intervention effects was not the objective of this study.

First, we will discuss results related to preconceptional FA knowledge. The inclusion of both men and women in our study enabled the evaluation of gender differences in knowledge. As expected, men had less correct knowledge about the need for preconceptional FA supplements. Corresponding results were found in another study conducted in a random sample of men and women in the US [21].

With regard to age differences, preconceptional FA knowledge was lower among the younger respondents (16–24 years) and the non-fertile age group (45 years and older) [22, 23]. For the younger age category, similar results have been found in other studies [24–26]. The number of teenage (<20 years) births is less than a half percent of all births in the Netherlands. However, the youngest age groups comprise of future parents and teenage mothers are still common among Surinamese and Antillean girls [27].

As expected, our results showed that individuals aged between 25 and 44 years showed the highest level of correct knowledge concerning preconception FA supplementation.

Respondents aged over 45 years were included in our study, because their knowledge and beliefs can influence behaviour of younger people. Studies showed that social influences (e.g. knowledge and habits) plays an important role in the development of behavior [28]. Approaches with social network and social influences have been successfully used for a range of health behaviors, including HIV risk practices, smoking, exercises, dieting, family planning, and mental health [29]. In addition, cultural diversity in social influences exists and is a variable that merits attention in health programs. We also found that preconception FA knowledge was lower amongst non-Western women. This may suggest that language proficiency within the host country has consequences for knowledge of preconception FA supplements [30, 31]. As expected and supported by other studies, irrespective of ethnic background, individuals with higher levels of education more often showed correct knowledge of preconception FA use [12, 26, 32]. Whether this also leads to actually behaviour change (e.g. preconception FA use) is unknown from our study. However, a recent study found a positive association between level of education and both knowledge and preconceptional FA supplementation [33]. This finding is in line with other studies revealing a gap with respect to FA use due to a difference in knowledge between women of different educational levels [34, 35].

Also in our study, the knowledge gap between non-Western immigrants and Dutch was significantly larger amongst individuals with lower levels of education. No apparent difference in knowledge was found among highly educated individuals. Different health and peer education programs have been conducted in the Netherlands to promote healthy behaviors amongst less educated non-Western immigrants [13, 36, 37]. One of the intentions in Ready for a Baby was to increase the low reproductive health (care) literacy of non-Western ethnic minority groups using targeted and customized peer education sessions [38]. These sessions were successful in reaching and educating non-Western ethnic minorities about reproductive behaviour e.g. utilization of FA supplements, and antenatal and postnatal care [38]. Besides knowledge transfer by educational sessions, nudging is a promising method which has generated great interest among policymakers [39]. Nudging suggests an approach to behaviour change that focuses on altering environmental cues to prompt healthier behaviour, e.g. making non-smoking more visible through mass-media campaigns, instead of regulating, e.g. ban smoking in public places.

In households without children, less correct knowledge about preconceptional FA use was observed. We expect that persons with children are more informed about FA use because of their previous contact with a GP, midwife or gynaecologist. In contrast with our results, other studies conducted in the Netherlands indicated that a previous pregnancy influenced FA knowledge and use of women in a negative way [12, 34].

Secondly, we will discuss the intention to seek out PCC. For this outcome a significant citywide increase was found from 16.1 % in 2007 to 24.6 % in 2012, followed by a temporary significant decrease. Within the two pilot districts a similar pattern was found. We assume that the intention to seek out PCC increased as a result of the interventions during the Ready for a Baby program (2008–2012), and decreased when the interventions stopped. This would support the hypothesis that one-time campaigns seem insufficient for structural (preconceptional) behaviour change and continuous attention is needed.

The results showed a clear SES gradient: correct preconception FA knowledge was low amongst the low SES group, while low SES showed higher levels of intention to consult a GP or midwife preconceptionally. We believe that these low SES respondents have less internal resources (e.g. education, health literacy, and language proficiency) to rely on their own abilities, which results in higher utilization of health care services (e.g. higher intentions to seek for PCC).

Our study has some limitations. First, our results may have been affected by non-response rates. Relative to the Dutch population, immigrants were less represented over the eight study years. However, we do not expect this to have affected our results for the following reason. The Amsterdam Born Children and their Development-study on ethnicity related perinatal health [40] examined the effect of selective ethnicity-related non-response. The study was able to pursue an empirical approach of non-response effects: data on non-respondents (outcomes and determinants) could be retrieved anonymously from national registries. It was observed that the prevalence of outcomes and determinants (like e.g. education) were affected due to selective participation. However, associations and results from regression analysis were not affected to any relevant degree. Second, although a questionnaire is a method for collecting information, misclassification or bias through language proficiency should always be considered.

Our study has several strengths. Firstly, it had large numbers of participants, including different ethnic and socio-economic groups, allowing us to evaluate a large number of covariates. Secondly, the study was not limited only to women of childbearing age and/or women who wish to fall pregnant, but was based on the general population in a large city in the Netherlands. This enhances the external validity of the findings. Finally, as it was not limited to women, we were able to evaluate gender differences.

## Conclusion

This study shows that knowledge on preconceptional FA supplementation is still too low, especially among low SES respondents. For the intention to seek for PCC a vice versa effect was found; namely the lower the SES, the higher the PCC intention. An one-time national public campaign with FA recommendations [34] did not manage to attain a permanent change in (preconceptional) behaviour and/or knowledge. Preconception health should be considered as a critical stage in the continuum pregnancy related care. We recommend a continuous approach to increase PCC knowledge among the general population. This approach has to go far beyond one-time campaigns or interventions including integration with other preventive health care maximizing demand for and uptake of preconception interventions, especially by adolescents and low socio-economic status groups. This could be realized through activities at school (e.g. integrating PCC in sexual health education at high schools), general health-education programs at school, or in health care center. Peer education seems to be a promising health promotion approach, which can be employed with regard to many subgroups that are now difficult to reach for the regular health care [38]. Finally, involvement of men in preconception health and health care use could be an opportunity to increase PCC uptake.
